# The Role of Situational Variables in Analysing Physical Performance in Soccer

**DOI:** 10.2478/v10078-012-0082-9

**Published:** 2012-12-30

**Authors:** Carlos Lago-Peñas

**Affiliations:** 1 Faculty of Education and Sports Sciences, University of Vigo, Av. Buenos Aires, Pontevedra, Spain.

**Keywords:** fatigue, soccer, physical performance, situational variables

## Abstract

Performance analysis in sport is used to investigate the performance of teams and players across different sports. Research within this area, especially when focussing on the determinants of success, has grown rapidly in the last few years. During this time, the role of a new concept, ‘situational variables’ has emerged. This term includes the different game and situational conditions that may influence performance at a behavioural level. Given that soccer is dominated by strategic factors, it is reasonable to suggest that situational variables of match status (i.e. whether the team is winning, losing or drawing), quality of opposition (strong or weak), and match location (i.e. playing at home or away) may somehow influence the teams’ and players’ activities. These situational variables need to be analyzed in depth to understand their influence in team sports. The aim of this article was to examine the independent and interactive effects of situational variables on physical performance in elite soccer. The view that professional soccer players regulate their physical efforts according to the specific demands of individual matches and periods of the game is offered. In support of this argument results from recent studies are presented. Implications of this perspective for match analyst and coaches for evaluating performance are also considered.

## Introduction

The physiological demands of soccer have been studied intensively in male players. Time – motion analysis research has demonstrated that elite players typically cover distances of 9 – 14 km during a match (Barros et al., 2007; Di Salvo et al., 2007; Mohr et al., 2003; Rampinini et al., 2009). The type of exercise in soccer is intermittent, with a change in activity every 4 – 6 s. (Bangsbo, 1994; Mohr et al., 2003). Thus, an international top-class player performs approximately 1330 activities during a match, including about 220 runs at high speed (Barros et al., 2007; Di Salvo et al., 2007; Mohr et al., 2003; Rampinini et al., 2009). Playing a high-level match can elicit up to 75% of maximal oxygen uptake, with the anaerobic system contributing greatly during intense periods (Bangsbo, 1994; Mohr et al, 2003). Several studies have shown decrements in physiological performance during matches. In particular, it has been suggested that high-intensity running and sprinting decrease from the first to the second half, probably due to physical fatigue (Barros et al., 2007; Mohr et al., 2005; Rampinini et al., 2007; 2009; Wisløff, 2009).

However, results from different recent studies using sophisticated measurement technologies emphasize the importance of accounting for the different game and situational conditions that may influence performance at a behavior level (Bradley et al., 2009; 2010; Di Salvo et al., 2009; Carling, 2010; Castellano et al., 2011; Lago-Peñas et al., 2009; Zubillaga et al., 2007). Given that soccer is dominated by strategic factors, it is reasonable to suggest that situational variables may somehow influence the teams’ and players’ activities. Empirical evidence suggests that the situational variables of match location (i.e. playing at home or away), match status (i.e. whether the team was winning, losing or drawing), and the quality of the opposition (strong or weak) are very important factors for soccer performances. According to different studies, the importance of these situational factors is reflected in changes in the teams’ and players’ activities as a response to match situations (Bloomfield et al., 2005a, 2005b; James et al., 2002; Jones et al., 2004; Lago and Martin, 2007; Lago-Peñas et al., 2009; Shaw and O’Donoghue, 2004; Taylor et al., 2008). It has been suggested that professional soccer players regulate their physical effort according to the specific demands of individual matches and periods of the game (Carling et al., 2008; Rampinini et al., 2007). However, despite the importance of accounting for match location, quality of opposition, and match status during the assessment of tactical aspects of soccer performance (Carling et al., 2005; Taylor et al., 2008), the effects of these situational variables on distance covered at various speed in elite soccer are still unclear. These situational variables need to be analyzed in depth to understand their influence in team sports.

The aim of this study was to examine the independent and interactive effects of situational variables on physical performance in soccer. The view that professional soccer players regulate their physical efforts according to the specific demands of individual matches and periods of the game is offered. In support of this argument results from recent studies are presented. Implications of this perspective for match analyst and coaches for evaluating performance are also considered.

## The effects of situational variables on physical performance

### Match Status

Performance accomplishments are a powerful source of efficacy expectations and such expectations determine the task-related effort that has to be expended (Bandura, 1977). In soccer, the match status may be viewed as a measure of performance accomplishments and hence, may influence the effort made by a player (O’Donoghue and Tenga, 2001). Match status is determined by whether a team or a player is winning, losing or drawing at the time a particular behaviour is recorded (Bloomfield et al., 2005a, 2005b; Taylor et al., 2008). According to Bloomfield et al. (2005a), Lago and Martin (2007) and Taylor et al. (2008), the importance of this situational variable is reflected in changes in team and players strategies in response to the score-line. Teams often show a more defensive strategy when winning than when losing, and vice versa. For low scoring team sports like soccer, there are just three major levels of match status to be considered during analysis (team winning, losing or drawing).

Existing notational analysis has provided information on the effects of match status on work rate in soccer. It has been demonstrated that soccer players perform significantly less high-intensity activity when winning than when losing or when the score is level (Bloomfield et al., 2005a, 2005b; Castellano et al., 2011; Shaw and O’Donoghue, 2004; O’Donoghue and Tenga, 2001). These results suggest that players do not always use their maximal physical capacity during the match. In fact, given that winning is a comfortable state for a team, it is possible that players assume a ball retention strategy, and slowing down the game resulting in lower speeds (Bloomfield et al., 2005b; Lago et al., 2010). On the other hand, when losing, players try to reach their maximal activity in order to win or draw the match. Other studies have considered match status in relation to the tactical aspects of performance. James et al. (2002) and Lago and Martin (2007) found that successful and unsuccessful teams had longer periods of possession in matches when they were losing than when they were winning. When ahead, teams decreased their possession, suggesting they preferred to play counter-attacking or direct play (that is, move the ball quickly to within scoring range, often using long passes or long balls downfield). However when behind, they increased their possession, suggesting they preferred to “control” the game by dictating play. In addition, Lago (2009) demonstrated that time spent in possession of the ball in different zones of the pitch (defensive third, middle third, attacking third) was influenced by match status: when teams were losing, possession of the ball was less in the defensive zone and more in the attacking zone than when winning or drawing. Finally, the relationship between match status and technical performances in team sports is still inconclusive. Taylor et al. (2008) found that playing at home and winning resulted in a decreased number of aerial challenges, dribbles, losses of control, passes, tackles, and times tackled, whereas the frequency of these behaviours increased when playing at home and losing. However, the outcomes of most behaviours were not influenced by the situation variables. Research is needed in order to clarify the effects of match status on technical performances.

### Quality of Opposition

Fatigue may be evident as a prolonged recovery during the game, for example increased time spent in low-intensity activities. The reason for this decline in performance could be repeated pressure from the opposition on an individual player, eventually leading to an inability to respond to game demands (Lago Peñas et al., 2009; Rampinini et al., 2007; Zubillaga et al., 2007). Rampinini et al. (2007) observed that the work rate of professional soccer players was significantly influenced by the activity profile of opponents. Fatigue during match-play comes and goes, with the player recovering once the pressure is off. When this happens, tactical support for the player is paramount, so hès not constantly under attack.. However, the same study (Rampinini, 2007), showed that the total distance covered and the amount of high-intensity running during matches were higher against `better’ opponent teams than against `weaker’ opponent teams. Other studies suggest that the poorer the quality of the opponent, the shorter the distance covered by the reference team (Bloomfield, 2005a; 2005b; Lago et al., 2010). These findings suggest that players can increase or decrease their work rate according to both demands of individual matches and to the quality of the opposition.

The opponent level has been considered from different methodological perspectives. For example, teams and players have been categorized as “successful” and “unsuccessful” according to their standings within a particular tournament or classified as “strong” or “weak” based on symmetric division of end-of-season classification (Taylor et al., 2008). Lago et al. (2010) defined the quality of opposition as the differences in the end-of-season ranking between opposing teams. Recently, team performance has been classified using cluster analysis procedures, which improved the classification by using more valid cut-off values.

### Game Location

Home advantage can be quantified as the proportion of all games that are won by the home team, or where ties are permitted, the proportion of all points. Several studies of the relationship between match location and work rate in soccer showed that home teams cover greater distances than away teams during low-intensity activity (Lago et al., 2010; Lago-Peñas et al., 2009; Zubillaga et al., 2007). Other studies suggest that the effect of this factor should be addressed in the interaction with other situational variables (e.g. playing at home and losing against a weak opponent) (Castellano et al., 2011). Despite the fact that home advantage in soccer is a well-known and well-documented fact (Brown et al., 2002; Clarke and Norman, 1995; Nevill and Holder, 1999; Pollard, 2008; Tucker et al., 2005), the precise causes and their simple or interactive effects on performance are still not clear. However, the most plausible explanations are: crowd effects, travel effects, familiarity, referee bias, territoriality, specific tactics, rule factors, and psychological factors (Pollard, 2008). Several authors have shown that in football shots, corners and other offensive performance measures follow a similar pattern to game outcome with regards to home advantage (Carmichael and Thomas, 2005; Poulter, 2007; Seckin and Pollard. 2008). However, similar effects for other measures such as offsides and fouls are less convincing. Tactics and strategies in football have also been investigated with significant differences found between home and away teams (Tucker et al., 2005).

### Interactive Effects

Existing notational analysis has provided preliminary information on the effects of situational variables such us match location, match status, and quality of the opponent on sports performance at a behavioural level. Nonetheless, with the exception of Lago and Martin (2007), Taylor et al. (2008), Lago (2009) and Castellano et al. (2011), previous research has examined situational variables independently not accounting for the possibility of higher-order interactions (e.g. playing at home and losing). However, the examination of situational variables in isolation would appear to provide limited insight into the complex nature of team sports performance (McGarry and Franks, 2003; Reed and O’Donoghue, 2005). Lago (2007) demonstrated variations in ball possession as a function of match location and match status, with home teams having more possession when drawing than away teams. Taylor et al. (2010) showed that the frequency of on-the-ball behaviours (passes, shots, tackles, clearances, crosses, dribbles, losses of control and aerial challenges) performed by a professional soccer team was explained by the interaction between such variables as match location and match status. In a recent work, Lago et al. (2010) examined the effects of match location, quality of opposition and match status on distance covered at various speeds in elite soccer. As can be seen in Table 1, physical performance was influenced by the situational variables, either independently or interactively. For example, the expected distance covered at maximal intensity (23 km·h^−1^) by players differs considerably according to match status, quality of opposition, and match location (by 31%).

## The influence of a congested calendar on physical performance in elite soccer

In modern professional soccer, the capacity to recover from intense training, competition and matches is often considered an important determinant of subsequent performance (Carling et al., 2012). Professional soccer players are often required to play competition matches with only 2–3 days’ recovery. In such conditions, the maintenance or improvement of the player’s activity is not only determined by appropriate conditioning but also by the ability of the body systems to recover and regenerate after multiple stress stimuli. Research has shown that participation in a soccer match results in muscle trauma and a reduction in the anaerobic performance of players with perturbations persisting up to 72 h (Ascensão et al., 2008; Ispirlidis et al., 2008). Therefore, one would expect a drop in measures of physical performance in competition when there is an insufficient recovery time between matches. Recently, several studies using sophisticated measurement technologies have analyzed the physical activity profiles and injury rates of English (Odetoyinbo et al., 2009), Spanish (Lago-Peñas et al., 2009; Lago-Peñas et al., 2010; Rey et al., 2010) and Scottish (Dupont et al., 2010) elite soccer players during intense periods of matches (2 or 3 consecutive matches played over 3.7 days time-scale). Unexpectedly, those studies did not reveal any statistical differences in the distances covered at various speeds across successive matches played in a short time.

Moreover, the incidence of match injury during the congested fixture period was similar to that reported in matches outside this period. However, Lago-Peñas et al. (2010) found the elite soccer players distance covered at various speeds during the congested fixture periods was dependent on match contextual factors. The top-class players performed less high-intensity activity (>19.1 km x h^−1^) when winning than when they were losing (p < 0.05), but more distance was covered by walking and jogging when winning (p < 0.05). The home teams covered a greater distance than away teams at low intensity (>14.1 km x h^−1^) (p < 0.01). Finally, the better the quality of the opponent, the higher the distance covered by walking and jogging.

These findings suggest that perhaps during a certain interval of time elite soccer players can cope physically even when the recovery time between matches is short. It has recently been suggested that professional soccer players regulate their physical efforts when participating in congested fixture periods (Castellano et al., 2011; Rampinini, 2007). While this self-imposition of efforts might partly explain the previously observed lack of a decline in physical performance during successive games with a short recovery period, no studies have verified this theory. Future studies should determine how long high-level performance can be maintained playing 2 matches a week.

## Conclusions

In conclusion, recent studies using sophisticated measurement technologies have demonstrated that the decrement in the total distance and in the distance covered at maximal intensity in the second half of the game is inconsistent. Professional soccer players regulate their physical efforts according to the specific demands of individual matches and periods of the game. Situational variables should be taken into account during the assessment of the physical aspects of soccer performance. As Taylor and coworkers (2008) explain, the implications for match analysts and coaches for evaluating performance and developing relevant training drills are very important. Existing recommendations suggest that the scouting of upcoming opposition should be carried out under circumstances that are reflective of the conditions under which the future match will occur (Kormelink and Seeverens, 1999). However, such procedures are unlikely to be practical due to time and resource constraints. Consequently, by establishing the impact of particular situational variables on performance, teams can be observed, when possible, with appropriate adjustments being made to analyses based on knowledge of such effects (Taylor et al., 2008). Similarly, post-match assessments of the technical, tactical, and physical aspects of performance can be made more objective by factoring in the effects of situational variables (Carling et al., 2005). Finally, if a notational analyst or coach has identified that the technical, physical or tactical aspects of performance are adversely influenced by specific situational variables, possible causes can be examined and match preparation focused on reducing such effects. Researchers should include situational variables in their explanatory models in order to better understand why physical demands vary throughout the game.

## Figures and Tables

**Table 1:** Simulated distance covered (m) at different speeds depending on match location, quality of opposition, and match status

		Home matches						Hawaimatches					
			
Match status	Quality of opposition	Total	0–11 km/h	11.1–14 km/h	14.1–19 km/h	19.1–23 km/h	>23 km/h	Total	0–11 km/h	11.1–14 km/h	14.1–19 km/h	19.1–23 km/h	>23 km/h
Winning 90 min	Strong	11140	7050	1744	1649	481	217	10856	6911	1584	1653	453	189
Winning 90 min	Weak	10824	6727	1662	1665	540	231	10540	6587	1501	1669	512	204
Losing 90 min	Strong	10856	6853	1678	1653	555	281	10641	6713	1518	1629	527	253
Losing 90 min	Weak	10540	6529	1596	1669	614	295	10325	6390	1435	1646	586	268
Drawing 90 min	Strong	11068	6990	1725	1632	496	225	10766	6890	1565	1646	468	197
Drawing 90 min	Weak	10802	6710	1654	1641	555	242	10441	6550	1505	1659	518	209

Source: Lago et al (2010).
